# New, Old, and Shared Antibody Specificities in Autoimmune Diseases

**DOI:** 10.3390/antib13010023

**Published:** 2024-03-13

**Authors:** Loredana Frasca, Anna Mennella, Raffaella Palazzo

**Affiliations:** National Center for Global Health, Istituto Superiore di Sanità, Viale Regina Elena 299, 00161 Rome, Italy; anna.mennella@iss.it (A.M.); raffaella.palazzo@iss.it (R.P.)

## 1. Introduction

Autoantibodies represent a primary characteristic of many systemic autoimmune diseases. In some autoimmune diseases, there are classes of antibodies that are more specific to that particular disease. For instance, in systemic sclerosis (SSc), a rare but severe autoimmune disease characterized by fibrosis of the skin and/or internal organs, there are specific autoantibodies called anti-centromere (ACA) or anti-topoisomerase (ATA) antibodies, which also distinguish the disease subtypes [1,2]. However, increasing evidence shows that autoantibodies can be also present in diseases that are not normally considered antibody-mediated; for instance, in two inflammatory skin diseases, psoriasis [3] (in which T-cells and innate immunity seem to play a major role) and atopic dermatitis (AD), there are autoantibodies [4,5]. In psoriasis, for example, autoantibodies can be present occasionally, and some of these autoantibodies can target LL37, an antimicrobial peptide (AMP) over-expressed in psoriatic lesional skin [3,6,7]. LL37 is known to be an autoantigen for T-cells of both the CD4 and CD8 phenotype in psoriasis [6].

Interestingly, in the arthritis associated with psoriasis, psoriatic arthritis (PSA), antibodies that also target post-translational modified autoantigens are present [7]. Anti-carbamylated or anti-citrullinated protein is present in PSA, including autoantibodies to LL37, which also recognize carbamylated and citrullinated LL37 (carb-LL37 and cit-LL37) [3,7]. Of note, anti-citrullinated protein antibodies (ACPAs), which are typically considered markers of rheumatoid arthritis (RA) [8], can be also detected in the blood of systemic lupus erythematosus (SLE) patients [9]. Interestingly, we described in SLE the presence of anti-LL37 antibodies and later we found that anti-cit-LL37 and anti-carb LL37 antibodies are also present in SLE [10,11,12]. Thus, these results have shown that anti-carbamylated and anti-citrullinated proteins are not only a feature of RA but are present in PSA, psoriasis, and SLE; this may suggest common mechanisms in the pathogenesis of these diseases.

Overall, these examples indicate that autoimmune diseases that seem to be different, as they present with different clinical manifestations, may share antibody reactivity.

Therefore, an understanding of the reasons why some antibodies mark only specific diseases whereas others are shared between diseases that affect different body locations could be very helpful in shedding light on common pathogenic pathways and, ultimately, repositioning pharmacological interventions. Moreover, the study of new, old, and shared antibody specificities across several autoimmune diseases can help in identifying more precise and distinct biomarkers in autoimmunity, even in less-studied autoimmune disorders.

By an extension of this field of studies, it is also interesting to study a different kind of antibody specificity detected in autoimmune (or autoinflammatory) diseases that is not directly linked to the type of auto-reactivity in that specific disease but induced as a consequence of a biological therapy. One of these examples concerns antibodies generated during anti-TNF-alpha therapy (anti-TNFs). Anti-TNFs have become a benchmark in the treatment of numerous autoimmune diseases: ankylosing spondylitis (AS), RA, hidradenitis suppurativa, Crohn’s disease (CD), polyarticular juvenile idiopathic arthritis, ulcerative colitis (UC), uveitis, PsA, and psoriasis [13,14]. Five anti-TNF agents are currently FDA-approved for the treatment of many joint- and gut-related inflammatory conditions: etanercept (composed of the TNFR2 fused to a human IgG1 Fc domain), infliximab (human–murine chimeric monoclonal IgG1 antibody), adalimumab (fully human antibody), certolizumab pegol (PEGylated Fab fragment of a humanised monoclonal antibody), and golimumab (fully human antibody) [13]. Although scientific efforts have been focused on antibody engineering in order to reduce the immunogenicity of biological drugs, the development of anti-drug-antibodies (ADAs) still forms the basis of the ineffectiveness or adverse reactions seen in a percentage of patients. Theoretically, ADAs are expected to affect treatment efficacy by lowering exposure to the free active drug via neutralization and/or enhanced clearance. When enough free drug is available to bind to its biological target, even when present, ADAs may not have clinical consequences [15].

Immediately after their introduction into the treatment protocols of several autoimmune and rheumatic diseases, scientific studies analyzed the immunogenicity of TNF inhibitors, showing a shorter drug survival in patients after subsequent doses of anti-TNFs [16]. Most anti-TNFs induce the formation of ADAs. As described above, TNF inhibitors are now widely used and have greatly improved medical care for patients. However, about 20% of psoriasis patients do not respond to treatment with TNF inhibitors, and around one third of initial responders lose their response over time [17]. Similar profiles have been observed for patients with RA and with inflammatory bowel disease (IBD) [18]. This limitation to the clinical efficacy of anti-TNF therapy can be explained by the immunogenicity potential of these drugs, considering that even humanized and fully human monoclonal antibodies can still induce ADA formation.

## 2. Overview of the Published Articles in the Special Issue “*New, Old, and Shared Antibody Specificities in Autoimmune Diseases*”

The first two contributions to our Special Issue are closely related and describe a new type of antibody found in SSc that targets chemokine (C-X-C motif) ligand 4 (CXCL4), also known as platelet factor 4, PF4 [19], a molecule highly upregulated in the blood and tissue of SSc patients [20,21]. SSc is a chronic disease characterized by skin/internal organ fibrosis, vasculopathy, and autoimmunity. CXCL4 is an early SSc biomarker that predicts worse disease outcome [20,21]. It was previously reported that CXCL4 acts as an autoantigen in SSc [22]. Anti-CXCL4 antibodies appeared in SSc blood and correlated with interferon-α (IFN-α). Such antibodies were more abundant in patients with interstitial lung disease (ILD).

### 2.1. Contribution 1

In this contribution, anti-CXCL4 antibodies were analyzed in an SSc cohort and put in relationship with anti-CXCL4 antibodies that recognize CXCL4 only in association with heparin. CXCL4 is a chemokine that can bind heparin and, on rare occasions, antibodies are induced (even in previously healthy individuals) that are specific to CXCL4-heparin complexes. These latter antibodies play a key role in heparin-induced thrombocytopenia (HIT), a rare condition in which they massively activate platelets, inducing dangerous thrombosis [23]. Since vasculitis and thrombosis are present in SSc, this paper addresses whether antibodies to CXCL4 in SSc are only directed to CXCL4 or also recognize complexes formed by CXCL4 and heparin. Heparin-dependent (HD) antibody titers were found to be higher in SSc compared to healthy subjects; interestingly, heparin-dependent antibodies appeared to be inversely correlated with the heparin-independent anti-CXCL4 antibodies, and the expression of the two antibody types was mutually exclusive in SSc blood. Heparin-dependent antibodies correlated with digital ulcers but did not correlate with IFN-α. This contribution represents a pilot study about these antibodies in SSc, to be replicated in wider SSc cohorts and in SSc subtypes.

### 2.2. Contribution 2

The second contribution exclusively studied anti-CXCL4 antibodies that are heparin-independent in SSc; in addition, it explored the presence of autoantibodies to CXCL4-L1, the non-allelic variant of CXCL4 differing from the wild-type CXCL4 by three amino acid substitutions at the COOH part of the molecule [19]. Short versions of CXCL4 and CXCL4-L1 mapping the 24 mer COOH part of the two molecules were used to distinguish between the two antibody specificities. The same antibody specificities were studied in VEDOSS (very early diagnosis of systemic sclerosis), separating SSc progressors from SSc non-progressors. Anti-CXCL4-L1-specific autoantibodies were especially detected in long-standing SSc (lsSSc), where they correlated with IFN-α. Thus, the data show that the early anti-CXCL4 autoantibody response qualitatively differs from a late response.

### 2.3. Contribution 3

The third contribution addresses a topic that is different but is still related to the antibody specificity found in autoimmune diseases. As specified in the introduction, currently, there are biological therapies for many autoimmune or auto-inflammatory diseases. Biological therapies include the use of antibodies on disease targets that have been found to be important players in the specific chronic diseases. As mentioned above, these treatments induce antibodies to the drug (ADAs). To avoid reactions from the patient’s immune system, especially to the Fc receptor of the antibodies used in therapy, researchers developed humanized antibodies [24,25,26]. However, this has not solved the problem. In this contribution, the authors describe a method of reducing the immunogenicity of antibodies used in therapy not only in autoimmune diseases but also for treating infectious diseases and hematological and solid tumor cancers [24,25,26].

The authors focus on the potential safety Issue linked to the Fc effector function. Fc silencing strategies have been characterized by the removal of the Fc glycosylation and point mutations introduced at specific locations in the lower hinge of the Fc [24]. Some strategies have relied on the low effector properties of IgG4 [27]. In this paper, the authors explore the possibility, less commonly considered, of deleting the hinge region. The removal of the hinge domain in humanized IgG1 and IgG4 mAbs blocked the ability to activate human Fc gamma receptors I and IIIA while preserving the capacity to engage target antigens.

### 2.4. Contribution 4

The authors of the forth contribution focused on natural antibodies (NAbs), a type of antibodies that are germ-line-encoded [28,29,30]. These kind of natural antibodies can show broad specificity for foreign but also self-antigens. Self-replenishing long-lasting B-cells produce them (B-1 cells) [28,29,30,31,32,33]. Many NAbs (>30%) recognize oxidation-specific epitopes (OSE) that are formed when there is oxidative stress [29,30,31]. NAbs recognize apoptotic cells, in part through the recognition of OSE on their cell membranes [29,34,35,36,37], and this is important to mediate the clearance of the apoptotic cells and their debris, a process that dampens inflammation. The persistence of debris is very important to promote inflammatory and autoimmune diseases [38]. By targeting OSE, as well as other epitopes, NAbs protect from atherosclerosis, several autoimmune diseases, and perhaps cancer. In this study, the authors aimed to further understand the CD1d-mediated regulation of B-1 cells secreting a type of NAb, E06 NAb, which recognizes OSE, including those present on LDL. E06 is an IgM NAb specific to phosphocholine (PC), the head group in oxidized phospholipids present on the surface of apoptotic cells and in oxidized LDL (OxLDL). They show that the titers of the E06 NAb strongly increased in Cd1d-deficient mice. The paper identifies, in a mouse model, a CD1d-mediated regulation of the E06 IgM-secreting cells. The authors conclude that the CD1d-mediated regulation of the E06 NAb generation is a novel mechanism that regulates the production of natural antibodies directed to oxidized epitopes.

### 2.5. Contribution 5

The last contribution also deals with SSc and specific autoantibodies. The article is an overview of studies on the association between SSc-specific antibodies and structural microvascular abnormalities evaluated using nailfold videocapillaroscopy (NVC), a method representing one of the diagnostic tools for SSc [1,2]. The authors present evidence of an association between typical SSc-specific autoantibodies and NVC results. ACA+ patients seem to show a slower microvascular and clinical progression compared with ATA+ and ARA+ (anti-RNA polymerase III) individuals. However, the same authors indicate that caution should be taken, as the data on ARA antibodies are more conflicting with regard to microvascular damage connections. The authors also suggest that the autoantibodies analyzed are not only an epiphenomenon but could mediate endothelial damage [39,40].

## 3. Conclusions

The contributions to this Special Issue all deal with antibodies or autoantibodies and their significance or relationship to autoimmune diseases. This collection provides a starting point for exploring in more details some of the aspects reported on. Three papers deal with SSc, a disease for which a cure is needed, as there are currently no effective therapies that block the disease.

Considering the conclusions of the review in contribution 5 and the two papers on anti-CXCL4 antibodies and SSc, it appears that only very large and multi-centric studies can address the relationships between autoantibodies that seem to be pathogenic for endothelial cells and NVC results in the prediction of severe disease. In this respect, we also believe that the anti-CXCL4 antibodies analyzed in contributions 1 and 2 are possibly worth analyzing in relation to NVC results, disease outcomes, and disease classification in larger and multiple SSc cohorts.

The study in contribution 4 on specific antibodies in germ-line shapes and the effect of the molecule CD1d could be also studied in autoimmunity.

For instance, pre-clinical models showed that the activation of the CD1d-iNKT cell axis could prevent or ameliorate established autoimmune diseases [41]. A critical intersection of the CD1d-iNKT cell axis with B-cells in healthy individuals and in patients with SLE also emerged from other work [42]. These antibodies could be better studied in SLE, for example.

Contribution 3 addresses the problem of anti-drug antibodies, a problem that can also be present in other pathological conditions treated with antibodies. The new generation of therapeutic mAbs in the clinical setting intends to enhance the beneficial effects of these agents while lowering the undesirable effects. Contribution 3 proposes the deletion of the hinge domain, with potential applications in the treatment of autoimmune diseases since this approach was already tested, with positive results, in rhesus monkeys with *myasthenia gravis* [27] and also in the inactivation of bacterial toxins during infection [43].
